# Supplementary material for: Loss of Function of the RRMF Domain in OsROS1a Causes Sterility in Rice (Oryza sativa L.)
Source: Int J Mol Sci. 2022 Sep 26;23(19):11349. doi: 10.3390/ijms231911349 (PMC9569698; doi:10.3390/ijms231911349)
Supplement: Supplementary file 1 [file ijms-23-11349-s001.zip › ijms-1905264-supplementary.pdf]

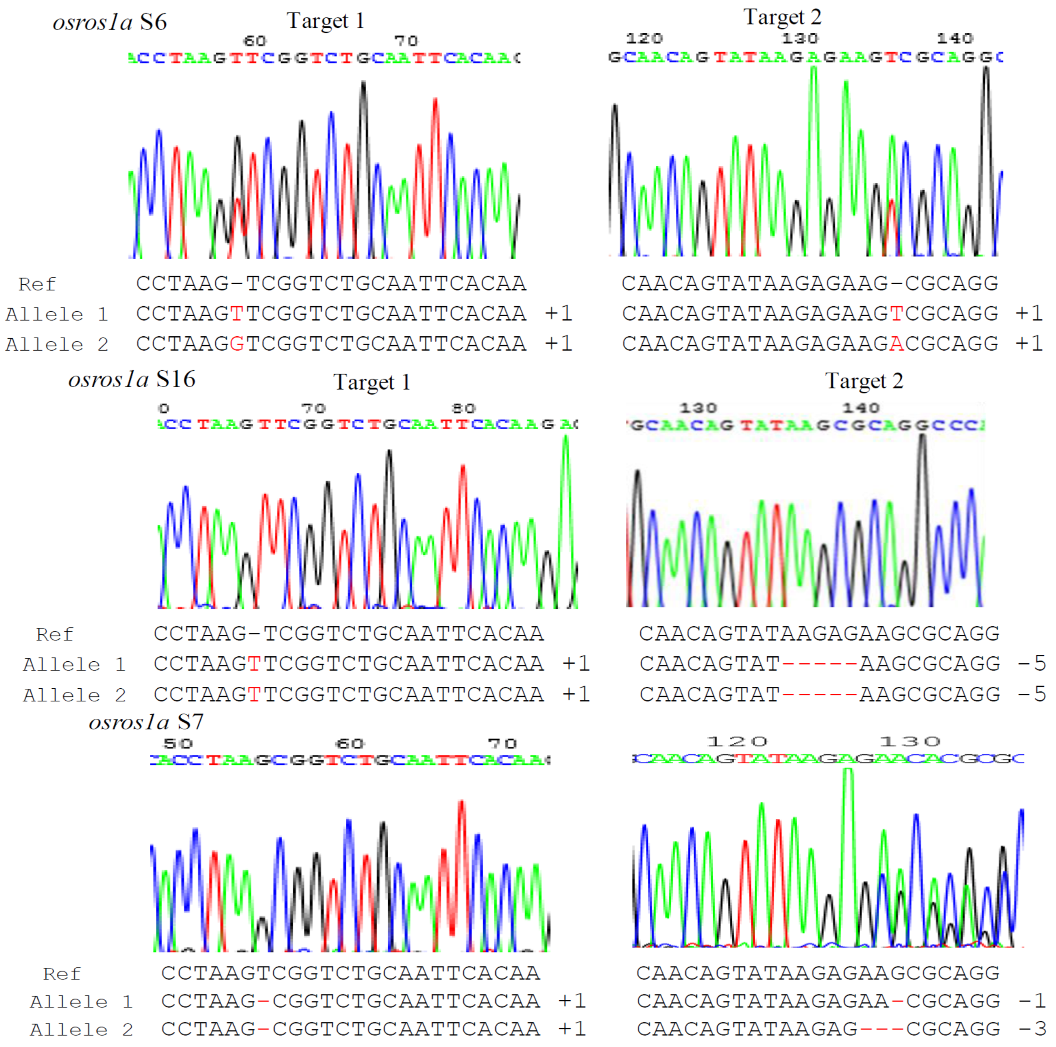

Figure S1. DNA sequencing chromatograms of *osros1a* mutants. Two targets were showed, and the indels in the target sites were showed red letters and dashes.

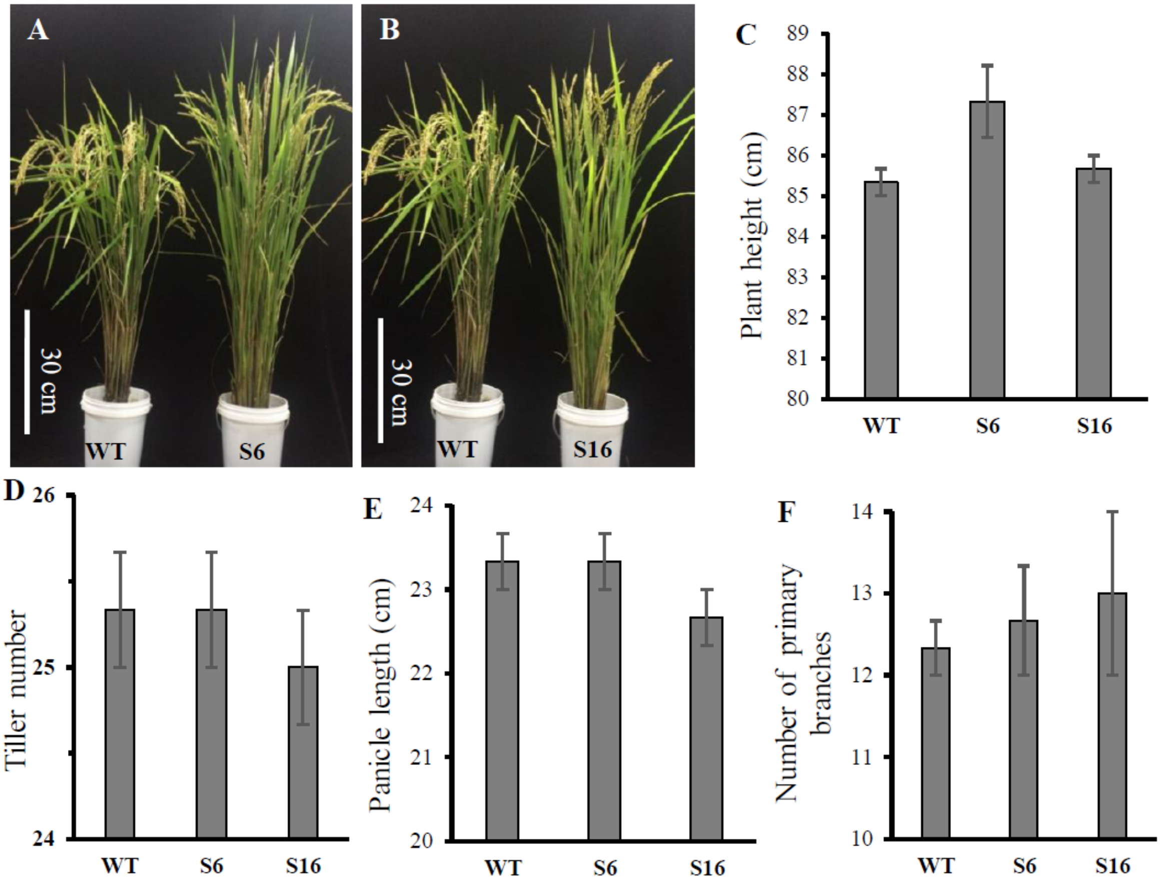

Figure S2. Phenotype comparison of the WT with *osros1a* mutants. The plant phenotypes of (A) WT and S6; (B) WT and S16. (C) Plant height, (D) Tiller number, (E) Panicle length and (F) primary branches number of WT, *osros1a* S6 and S16 mutants. Data are means ± SD (n = 3).

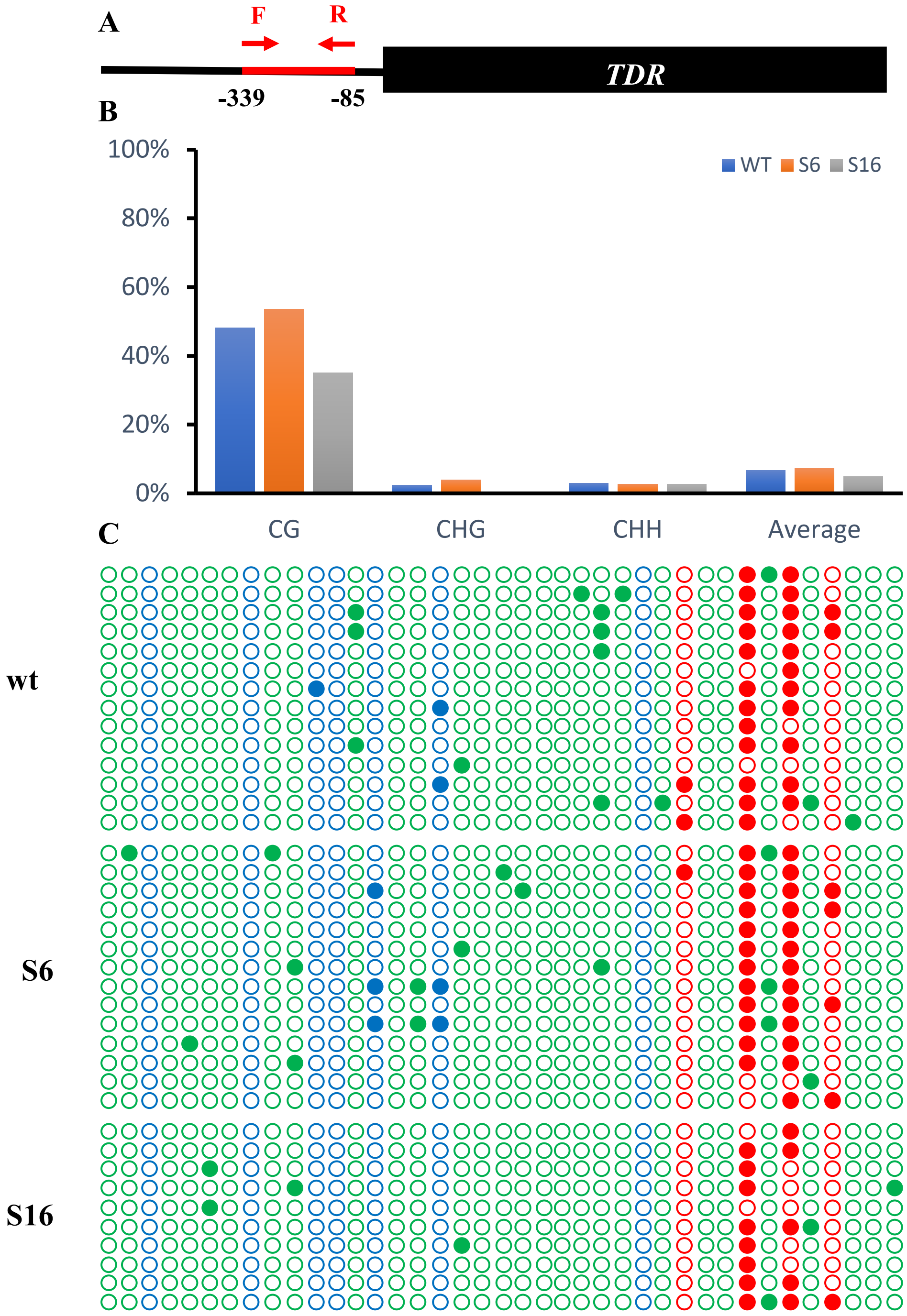

Figure S3. The DNA methylation of *TDR* gene promoter in young panicle. (**A**) The diagram of bisulfite sequencing of 225-bp *TDR* promoter. (**B**) The statistics of bisulfate sequencing of 225-bp *TDR* promoter in WT and *osros1a* mutants. (**C**) The dot plot comparison of DNA methylation between WT and *osros1a* mutants.

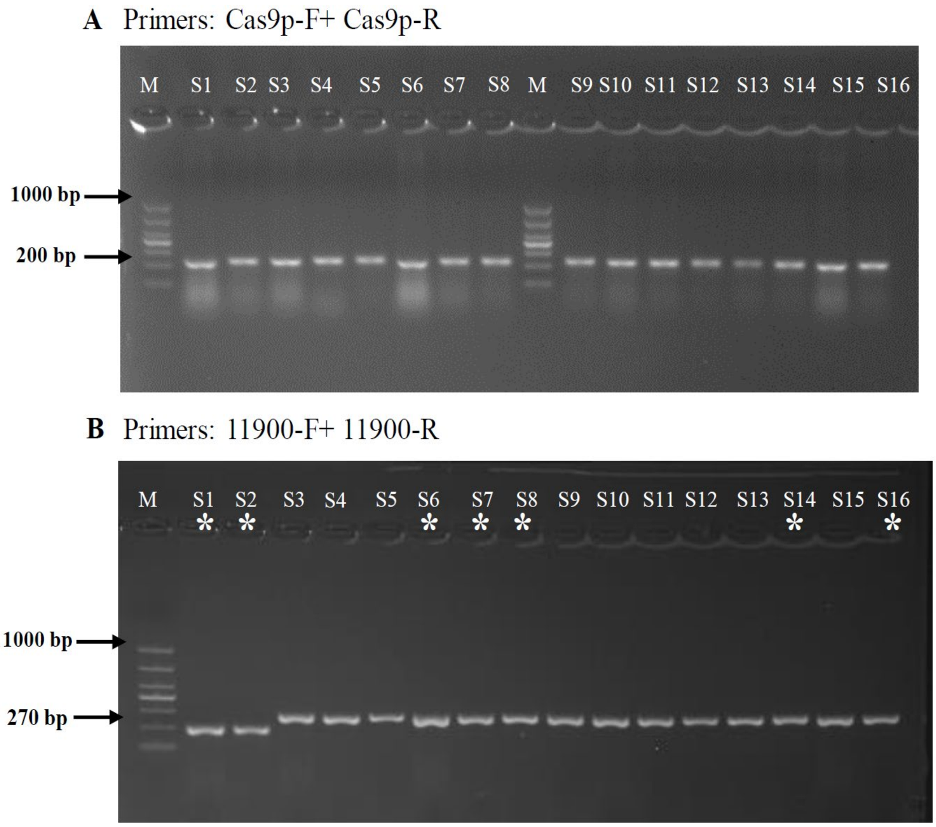

Figure S4. PCR of *OsROS1a* gene editing plants (A) with Cas9 primers and (B) with knockout target-specific primers. The white asterisk denotes the *osros1a* mutant lines.

Supplementary Table S1. Primers used in this study.

OSU6a promoter	
sg 11900.1F	GCCGTGTGAATTGCAGACCGACTT
sg 11900.1R	AAACAAGTCGGTCTGCAATTCACA
OSU6b promoter	
sg 11900.2F	GTTGCAACAGTATAAGAGAAGCGC
sg 11900.2R	AAACGCGCTTCTCTTATACTGTTG
pollen and anther related gene expression	
Actin F	CGTATGAGCAAGGAGATCAC
Actin R	CACATCTGTTGGAAGGTGCT
OsROS1a -F	ATCCTTGCCCATACCTAC
OsROS1a -R	AGCAGTGTCCCTCGAACT
TDR F	GGAGGTGGCACCAGTTTG
TDR R	AGGAGTCCAGTGGGATTGA
OsPKS2 F	ACTCTGTACCGTACCTGATCCA
OsPKS2 R	GTCACCGCCTTGTTGGAGATG
CYP704B2 F	CGACATGCCTTTCACCTCCT
CYP704B2 R	TGGAGGCAAACTCGAAGCTC
CSA F	AGAACTGGAACCTCATCGCC
CSA R	GACGTGCCAGTGGTTCTTGA
OspPGM-F	CTGCTCAGATTATCACTAAAATTG
OspPGM-R	AGAGCTAGGTCTATTAAAGGCTTC
BZR1 F	CCACCTACCGCAAGGGATG
BZR1 R	GTATCGTGTCCGGGTGCT
Bisulfite	
OsPKS2-BS- F	TAAAGTTATTAGAGGTGAGGGTTTG
OsPKS2-BS- R	AACCCTAAAAAAAATTAAAATAAATATC
TDR-BS-F	TGGAGAGGGATTAGATTAGTTTAAGG
TDR-BS-R	ATAAAAATTTTCTAACTCCAATACAAA
